# A conserved annexin A6–mediated membrane repair mechanism in muscle, heart, and nerve

**DOI:** 10.1172/jci.insight.158107

**Published:** 2022-07-22

**Authors:** Alexis R. Demonbreun, Elena Bogdanovic, Lauren A. Vaught, Nina L. Reiser, Katherine S. Fallon, Ashlee M. Long, Claire C. Oosterbaan, Michele Hadhazy, Patrick G.T. Page, Prem Raj B. Joseph, Gabrielle Cowen, Alexander M. Telenson, Ammaarah Khatri, Katherine R. Sadleir, Robert Vassar, Elizabeth M. McNally

**Affiliations:** 1 Center for Genetic Medicine,; 2 Department of Pharmacology, and; 3 Division of Cardiology, Department of Medicine, Feinberg School of Medicine, Northwestern University, Chicago, Illinois, USA.; 4 WuXi Biology, WuXi AppTec, Cranbury, New Jersey, USA.; 5 Department of Neurology, Feinberg School of Medicine, Northwestern University, Chicago, Illinois, USA.

**Keywords:** Cardiology, Muscle Biology, Muscle, Neurological disorders

## Abstract

Membrane instability and disruption underlie myriad acute and chronic disorders. *Anxa6* encodes the membrane-associated protein annexin A6 and was identified as a genetic modifier of muscle repair and muscular dystrophy. To evaluate annexin A6’s role in membrane repair in vivo, we inserted sequences encoding green fluorescent protein (GFP) into the last coding exon of *Anxa6*. Heterozygous *Anxa6gfp* mice expressed a normal pattern of annexin A6 with reduced annexin A6GFP mRNA and protein. High-resolution imaging of wounded muscle fibers showed annexin A6GFP rapidly formed a repair cap at the site of injury. Injured cardiomyocytes and neurons also displayed repair caps after wounding, highlighting annexin A6–mediated repair caps as a feature in multiple cell types. Using surface plasmon resonance, we showed recombinant annexin A6 bound phosphatidylserine-containing lipids in a Ca^2+^- and dose-dependent fashion with appreciable binding at approximately 50 μM Ca^2+^. Exogenously added recombinant annexin A6 localized to repair caps and improved muscle membrane repair capacity in a dose-dependent fashion without disrupting endogenous annexin A6 localization, indicating annexin A6 promotes repair from both intracellular and extracellular compartments. Thus, annexin A6 orchestrates repair in multiple cell types, and recombinant annexin A6 may be useful in additional chronic disorders beyond skeletal muscle myopathies.

## Graphical abstract

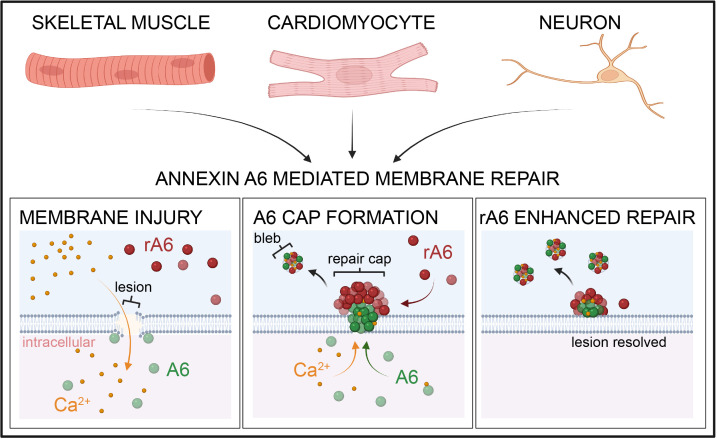

## Introduction

The plasma membrane is frequently exposed to mechanical disruption resulting in membrane lesions, which may vary in shape and size depending on cell type and function. Mutations in genes that function to either stabilize or repair the plasma membrane are associated with multiple distinct conditions, including muscular dystrophy, cardiomyopathy, and neuropathy (1, 2).

With membrane breach, the influx of extracellular Ca^2+^ can initiate plasma membrane repair. In the case of small membrane lesions, several models of plasma membrane repair have been proposed (3–5). In one model, intracellular vesicles are recruited to the site of injury in a Ca^2+^-dependent manner, where they fuse with each other and to the membrane lesion (6–8). A second model implicates constriction of the injured membrane followed by budding and shedding of the injured membrane into the extracellular space (9–12). Others have described endocytosis of the injured membrane area in addition to lateral diffusion of membrane to the site of injury (13–16). Aspects of each of these models may ultimately contribute to membrane repair and resealing and, in part, be dependent on cell type and the size and depth of the membrane disruption. The machinery that mediates membrane repair participates in other cellular transport processes, scaffolding receptor and signaling complexes, and modulating actin dynamics, which are not exclusively dedicated to membrane repair.

Annexin proteins bind to negatively charged phospholipids in a Ca^2+^-dependent manner and have been recognized for their role in membrane repair, in addition to cell migration and adhesion (17–22). In the setting of muscle membrane repair, a repair cap forms at the site of injury. Annexins A1, A2, and A6 are recruited to the repair cap in a Ca^2+^-dependent manner (23–26). Annexins A6 and A2 have the highest affinities for Ca^2+^, being recruited to the membrane lesion within 1–2 seconds of injury, followed by a second wave of annexin translocation including annexins A1, A4, and A5 (24, 25, 27). Annexins A6 and A2 are highly relevant for muscle membrane repair, while annexin A1 appears more dispensable (28–30). The differential response of annexin family members to intracellular Ca^2+^ levels allows for a graded and reinforced injury response.

Efficient sarcolemma repair is also critical for cardiomyocyte survival because these cells are terminally differentiated and have limited capacity for self-regeneration. Annexin A5 and annexin A6 are the most abundantly expressed annexin proteins in the heart (31). In mice, administration of recombinant annexin A5 reduces infarct size and preserves cardiac function after myocardial ischemia/reperfusion, demonstrating a cardioprotective effect of annexin A5 (32). The role of annexin A6 in mediating plasma membrane repair in cardiomyocytes was not evaluated in mice lacking *Anxa6* (encoding annexin A6) or in mice overexpressing *Anxa6* in the heart (33, 34).

As in skeletal and cardiac muscle, mechanical stress results in plasma membrane disruption of neurons (35, 36). Disruption of axonal membranes is an early event following traumatic brain injury in humans and experimental animal models of traumatic brain injury (37, 38). Similar to repair of skeletal muscle, repair of neuronal membrane after mechanical disruption is dependent on calcium and actin cytoskeletal dynamics (36). After spinal cord injury, annexin A1, A2, and A5 expression is upregulated 3 to 28 days postinjury with increased localization in neuron and glial cells; annexin A6 was not evaluated (39). Additionally, loss of annexin A1 increases permeability of the blood-brain barrier, which was rescued with recombinant annexin A1 administration (40, 41). Moreover, a protective role for annexin A5 has been identified in maintaining the blood–cerebrospinal fluid barrier (42). Plasmid overexpression of annexin A6 showed that annexin A6 localizes to the plasma membrane of injured neuroblastoma cells in culture, further suggesting that the mechanisms regulating muscle membrane repair may also occur in the brain (43).

We evaluated the in vivo role of annexin A6 in plasma membrane repair by using CRISPR/Cas9 to engineer a green fluorescent protein (GFP) tag at the carboxy terminus of annexin A6, termed annexin A6GFP. In this model, annexin A6 expression is driven from the *Anxa6* gene locus and it is not overexpressed. When subjected to plasma membrane injury, genomically encoded annexin A6GFP formed time-dependent repair caps in skeletal muscle, cardiomyocytes, and neurons. Exogenously added recombinant annexin A6, labeled with an alternative fluorescent tag, targeted the endogenous repair cap at the site of membrane injury in muscle and neurons. Together, these data support a general model of cellular repair for which recombinant annexin A6 may be a useful resealing agent.

## Results

### Generation of Anxa6gfp mice using gene editing.

**Figure 1:**
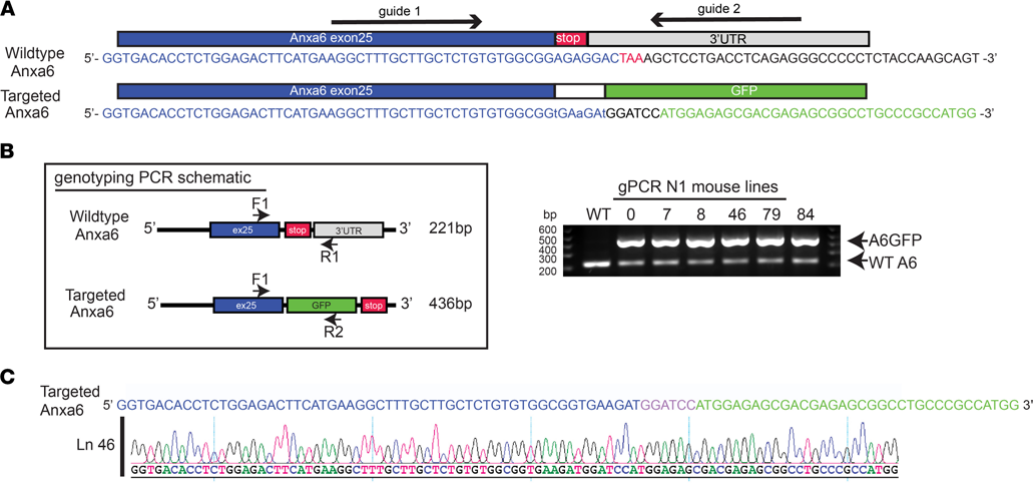
Generation of genomically encoded annexin A6GFP using CRISPR/Cas9 genome editing. (**A**) Targeting strategy for generating genomically encoded annexin A6GFP at the endogenous annexin A6 locus. Red lettering indicates protospacer adjacent motif sequence. Lowercase lettering indicates synonymous mutations in targeted allele. (**B**) *Anxa6gfp* mouse generation strategy. Genotyping schematic and PCR screening of *Anxa6gfp* of 6 heterozygous N1 offspring lines. (**C**) Representative sequence chromatograms of in-frame GFP insertion into the annexin A6 locus in *Anxa6gfp* CRISPR/Cas9-edited mouse line 46.

**Figure 2:**
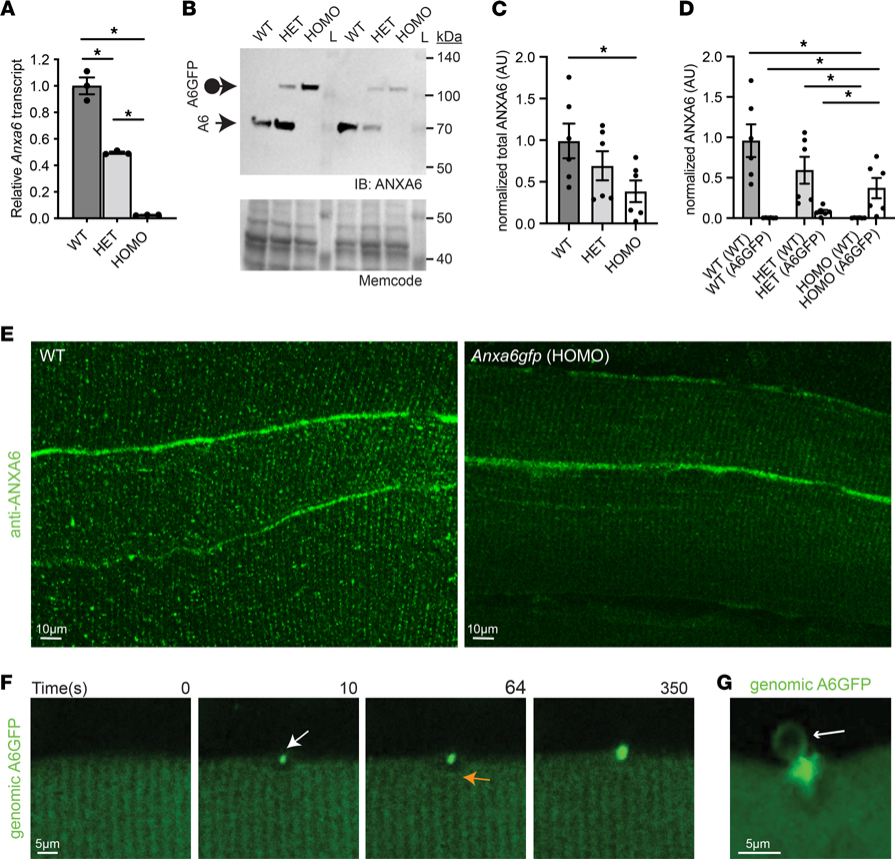
Genomic A6GFP protein localizes to the site of muscle membrane injury. (**A**) Quantitative PCR demonstrates reduced *Anxa6* levels in quadriceps from heterozygous and homozygous *Anxa6gfp* mice compared with wild-type (WT) controls. (**B**–**D**) Anti–annexin A6 immunoblots demonstrate reduced ANXA6 protein levels in quadriceps muscles from heterozygous and homozygous *Anxa6gfp* mice. The loading control is a 42 kDa band detected by MemCode reversible protein stain. (**E**) Anti–annexin A6 (shown in green) immunofluorescence imaging of extensor digitorum longus myofibers from WT and homozygous *Anxa6gfp* mice. ANXA6 and annexin A6GFP protein localize in a similar punctate, sarcomeric pattern and at the sarcolemma. Scale bar: 10 μm. (**F**) Upon laser-induced membrane injury, annexin A6GFP localized to the repair cap (white arrow) with a visible clearance zone (orange arrow) beneath the membrane lesion in heterozygous *Anxa6gfp* myofibers. (**G**) Genomically encoded annexin A6GFP membranous blebs (white arrow) erupt from the site of membrane injury. *Z*-stack images from an injured myofiber. Scale bar: 5 μm. *n* = 6 mice per genotype. *n* > 10 myofibers. *P < 0.05 by 1-way ANOVA.

CRISPR/Cas9 was used to replace the stop codon in the last exon of the *Anxa6* locus with sequences encoding GFP (Figure 1, A–C). A dual-guide strategy was used to insert the GFP-encoding sequences into embryonic stem cells, which were subsequently injected into blastocysts to create founder mice (Figure 1, A–C). This strategy mirrors the carboxy terminal GFP tags used in annexin A6 plasmid-mediated overexpression studies. We characterized both heterozygous and homozygous *Anxa6gfp* mice. Quantitative PCR analysis documented a reduction in *Anxa6* transcript in heterozygous mice compared with wild-type and further reduction in homozygous mice (Figure 2A). An amino-terminal antibody specific to annexin A6 was used to evaluate both endogenous annexin A6 and genomically encoded annexin A6GFP protein expression in quadriceps muscles by immunoblot (Figure 2B; see complete unedited blots in the supplemental material). Genomically encoded annexin A6GFP protein was reduced compared with endogenous annexin A6, such that heterozygous *Anxa6gfp* mice expressed 69% of the total annexin A6 protein levels expressed in wild-type quadriceps muscles, while homozygous *Anxa6gfp* mice expressed 38% of the total annexin A6 protein levels expressed in wild-type quadriceps muscles (Figure 2C). Of the reduced quantity of annexin A6 expressed in heterozygous *Anxa6gfp* muscle, 11% was annexin A6GFP protein (Figure 2D). To confirm that annexin A6GFP localized in a similar pattern to endogenous ANXA6 protein in myofibers, extensor digitorum longus myofibers were isolated from wild-type and homozygous *Anxa6gfp* mice and stained with anti-ANXA6 antibody. Homozygous mice were chosen as a comparator, as all annexin protein expressed is annexin A6GFP. Immunofluorescence microscopy showed that ANXA6 and annexin A6GFP localized in a similar punctate, sarcomeric pattern and at the sarcolemma (Figure 2E). The reduced fluorescence intensity was probably reflective of the reduced level of annexin A6GFP protein (Figure 2B). To verify that annexin A6GFP, which was expressed at lower than wild-type annexin A6 levels, localized to the site of membrane injury, we imaged flexor digitorum brevis myofibers isolated from *Anxa6gfp* mice. Within seconds of laser-mediated injury, genomically encoded annexin A6GFP localized to the membrane lesion, organizing into a repair cap (Figure 2F and Supplemental Video 1; supplemental material available online with this article; https://doi.org/10.1172/jci.insight.158107DS1). High-magnification imaging revealed the presence of annexin A6GFP–containing membranous blebs emanating from the repair cap (Figure 2G). These data combined show that *Anxa6gfp* mice express genomically encoded annexin A6GFP protein in the expected pattern in muscle but with reduced annexin A6GFP expression compared with endogenous annexin A6.

### Normal muscle in Anxa6gfp mice with normal muscle repair.

Prior studies have shown minimal phenotype in *Anxa6-*null mice, with no discernible impact on any of the major organs including skeletal muscle, heart, and brain (44). Similarly, annexin A6GFP protein expression in heterozygous and homozygous mice resulted in no overt muscle, heart, or brain defects in the background of otherwise healthy mice. Specifically, we detected no immune infiltrate, fibrosis, or internal nuclei, and histologically, normal muscle was indistinguishable from annexin A6GFP–expressing muscle (Supplemental Figure 1A). In both heterozygous (Figure 2F) and homozygous (Supplemental Figure 1B) *Anxa6gfp* myofibers, genomically encoded annexin A6GFP localized to a repair cap at the site of laser-induced injury. Myofibers were incubated in FM 4-64, a fluorescent indicator dye that increases fluorescence intensity upon insertion into injured membranes. Homozygous *Anxa6gfp* myofibers had similar levels of FM dye uptake compared to wild-type myofibers, showing intact muscle repair capacity (Supplemental Figure 1C). Thus, the GFP tag on annexin A6 did not detectably affect the repair response of annexin A6 in muscle, consistent with prior studies in which annexin A6GFP was expressed from a plasmid.

### Genomically encoded annexin A6GFP forms a repair cap with annexins A1 and A2 at the site of muscle membrane injury.

Electroporation of annexin-encoding plasmids was previously used to demonstrate annexin A6 forming a repair cap with annexins A1 and A2, and these studies relied on annexin overexpression. To determine whether genomically encoded annexin A6GFP expressed at levels lower than wild-type could still nucleate the annexin repair complex at the site of injury, *Anxa6gfp* myofibers were electroporated with annexin A6-tdTomato, annexin A2-tdTomato, or annexin A1-tdTomato plasmid and subjected to laser-induced injury. Genomically encoded annexin A6GFP localized to the site of injury, colocalizing with annexin A6-tdTomato, annexin A2-tdTomato, and annexin A1-tdTomato (Supplemental Figure 2). Genomically encoded annexin A6GFP protein translocated normally to the site of injury, colocalized with other annexins at the site of injury, and was associated with WT levels of FM 4-64 area after injury; these data support normal function for the GFP-tagged, genomically encoded annexin A6 in membrane repair (Supplemental Figures 1 and 2).

### Annexin A6GFP is expressed in the heart and forms repair caps in injured cardiomyocytes.

**Figure 3:**
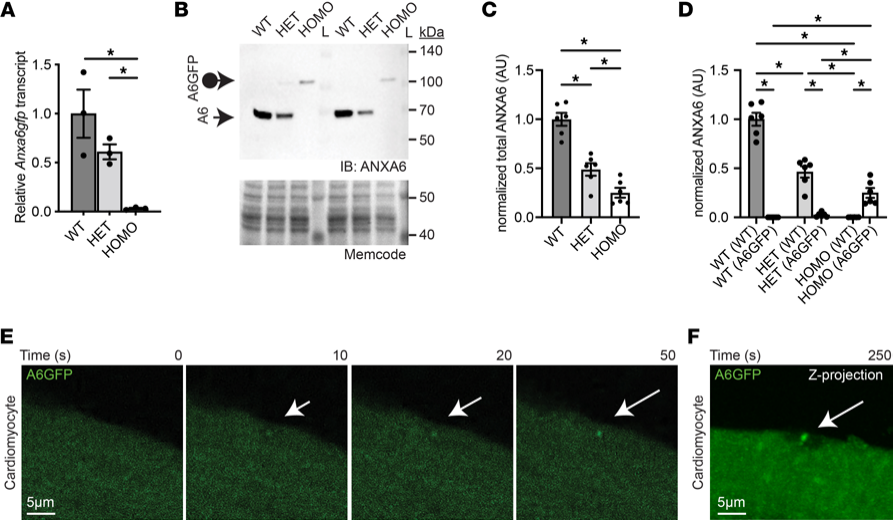
Genomically encoded annexin A6GFP localizes at the site of cardiomyocyte membrane injury. (**A**) Quantitative PCR demonstrates reduced *Anxa6* levels in heart lysates from heterozygous and homozygous *Anxa6gfp* mice compared with WT controls. (**B**–**D**) Anti–annexin A6 immunoblots demonstrate reduced ANXA6 protein levels in cardiac ventricle lysates from heterozygous and homozygous *Anxa6gfp* mice. The loading control is a 42 kDa band detected by MemCode reversible protein stain. (**E**) Adult ventricular cardiomyocytes were isolated from homozygous *Anxa6gfp* mice and subsequently laser-damaged. annexin A6GFP (shown in green) quickly localizes to the cardiomyocyte repair cap (white arrow). (**F**) *Z*-projection of a homozygous *Anxa6gfp* cardiomyocyte illustrating annexin A6GFP repair cap (white arrow) above the annexin-free zone at the site of injury 250 seconds after cardiomyocyte wounding. Scale bar: 5 μm. *n* = 6 mice per genotype. *n* > 12 cells from 5 isolations. *P < 0.05 by 1-way ANOVA.

The degree to which annexin A6–containing membrane repair complexes are found outside of myofiber repair is not known. As in skeletal muscle, a reduction in cardiac *Anxa6* transcript level in heterozygous and homozygous mice was documented utilizing quantitative PCR analysis (Figure 3A). Immunoblot analysis with an N-terminal anti–annexin A6 antibody supported a reduction in ANXA6 protein in *Anxa6gfp* heterozygous (49%) and homozygous cardiac ventricular lysates (25%) compared with wild-type ANXA6 protein levels (Figure 3, B and C; see complete unedited blots in the supplemental material). Of the reduced quantity of annexin A6 expressed in heterozygous *Anxa6gfp* cardiac ventricular lysates, 5% was annexin A6GFP protein (Figure 3D). Since skeletal myofibers and cardiomyocytes share many structural and functional features, we isolated and injured cardiomyocytes from *Anxa6gfp* mice to evaluate cardiomyocyte membrane repair (Figure 3E). *Anxa6gfp* ventricular cardiomyocytes were isolated, and the laser injury protocol was modified for use on cardiomyocytes. Compared with skeletal myofibers, cardiomyocytes were exquisitely sensitive to laser injury. Accordingly, the laser power was reduced by approximately 50% and external calcium levels were reduced 50% (500 μM) to accommodate this increased sensitivity to injury. Annexin A6GFP was observed in a sarcomeric pattern in live cells with some diffuse cytoplasmic fluorescence, in a pattern consistent with the known localization of cardiac annexin A6 (Figure 3F) (24, 45). Within 10 seconds of laser-wounding, annexin A6GFP localized to the membrane lesion, organizing into a repair cap in the cardiomyocyte (Figure 3E). Time-lapse images illustrate the progression of annexin A6GFP localization into the repair cap (arrow) in an isolated cardiomyocyte through 50 seconds postinjury (Figure 3E and Supplemental Video 2). A *Z*-projection image taken 250 seconds after laser injury depicts a repair cap above the annexin-free zone of an injured cardiomyocyte, similar to the repair structure seen in myofibers (Figure 3F). Thus, genomically encoded annexin A6GFP localized to the site of membrane injury, forming a repair cap at the membrane lesion in live adult ventricular cardiomyocytes, consistent with a conserved repair response of annexin A6 between cardiomyocytes and skeletal myofibers.

### Annexin A6 localizes to neuronal membrane lesions.

**Figure 4:**
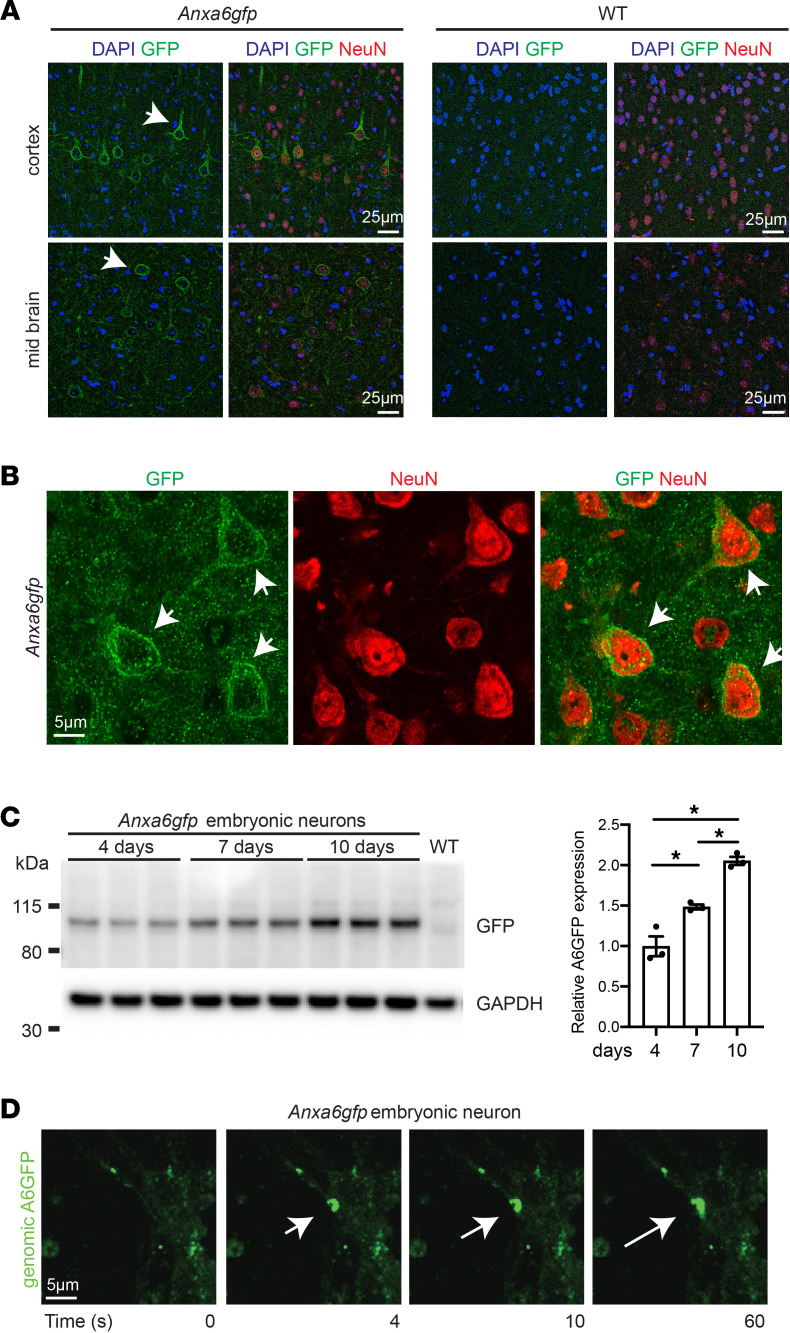
Annexin A6GFP localizes at the site of neuron membrane injury. (**A**) Anti-GFP (shown in green, indicated by arrows) antibody detects genomically encoded annexin A6GFP protein in *Anxa6gfp* adult cortex and midbrain but not in WT mice. DAPI (blue) marks nuclei. Anti-NeuN (red) marks mature neurons. (**B**) Anti-GFP (green, arrow) antibody, which detects genomically encoded annexin A6GFP protein, localized to the peripheral membrane of NeuN^+^ cortical neurons as visualized with high-magnification confocal imaging. (**C**) Embryonic neurons were isolated from *Anxa6gfp* mice. Genomically encoded annexin A6GFP expression increases with maturation. After 10 days, neurons expressed 2-fold more annexin A6GFP than at 4 days. (**D**) Isolated neurons were injured with a confocal laser. Genomically encoded annexin A6GFP (green) quickly localized into a repair cap (white arrow) visible 4 seconds postinjury. Multiple cells from *n* = 3 mice. *P < 0.05 by 1-way ANOVA.

Using the *Anxa6gfp* mouse model, we evaluated whether endogenous annexin A6 translocation was a component of primary neuronal cell injury repair. Brain imaging of *Anxa6gfp* mice using anti-GFP antibodies detected annexin A6GFP protein, largely restricted to the plasma membrane, and this was well seen in cortical neurons, as marked by NeuN positivity (Figure 4, A and B). Neurons were isolated at embryonic days 15–16 and cultured under maturation conditions. As neuron maturation progressed from day 4 to day 10 in culture, annexin A6GFP levels significantly increased (Figure 4C; see complete unedited blots in the supplemental material). Day 7 neurons were subjected to laser-induced membrane injury. Genomically encoded annexin A6GFP localized to the site of neuron injury, forming a repair cap visible within 1–2 seconds of injury, which persisted through the 60 seconds of imaging (Figure 4D and Supplemental Video 3). Together, these findings show a similar translocation of annexin A6 to the site of injury in skeletal myofibers, cardiomyocytes, and neurons.

### Recombinant annexin A6 binds phosphatidylserine in a calcium-dependent manner.

**Figure 5:**
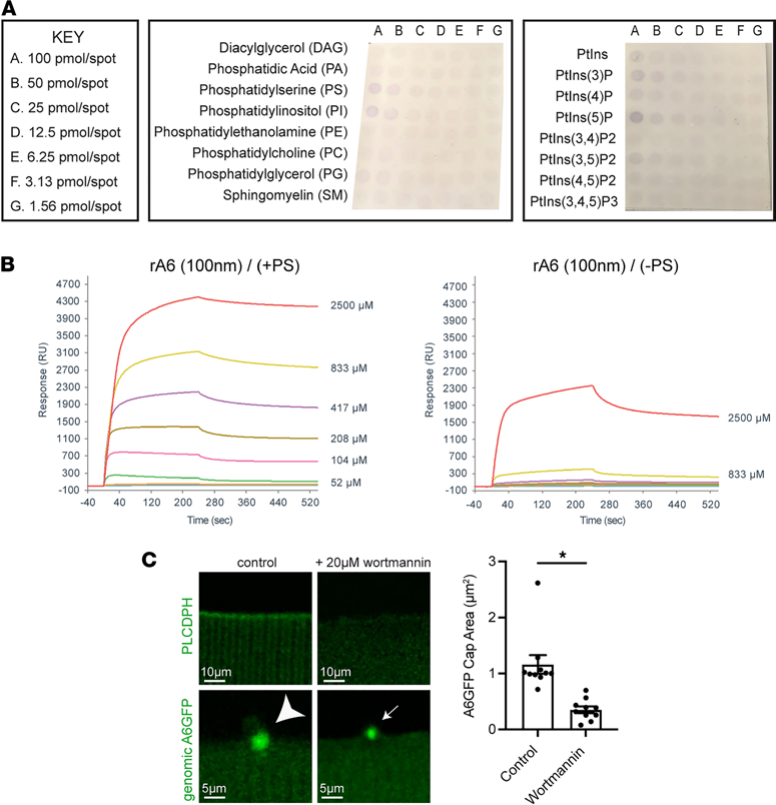
Recombinant annexin A6 binds PS. (**A**) Recombinant annexin A6 preferentially bound PS and phosphatidylinositol 5-phosphate on membrane lipid arrays. (**B**) SPR sensorgrams showing that recombinant annexin A6 (rA6) binds PS-containing liposomes at approximately 100 nM over a range of Ca^2+^ concentrations ranging from 0 to 2500 μM (0, 25, 52, 104, 208, 417, 833, and 2500 μM). (**C**) Wortmannin treatment depleted PIP2 in myofibers as visualized by reduced PLC-PH-EGFP signal (top panel). Additionally, wortmannin treatment reduced genomically encoded annexin A6GFP cap area after laser-induced injury. (*n* = 10 from 5 isolations; *P < 0.002 by *t* test).

Annexins are known calcium-dependent phospholipid binding proteins. Phosphatidylserine and phosphatidylethalomine are membrane lipids that normally are found in the inner plasma membrane leaflet and upon membrane injury flip to the outer leaflet. To evaluate phospholipid binding preference in vitro, recombinant annexin A6 was incubated on lipid arrays containing the common membrane lipids diacylglycerol, phosphatidic acid, phosphatidylserine (PS), phosphatidylinositol (PI), phosphatidylethanolamine (PE), phosphatidylcholine (PC), phosphatidylglycerol, and sphingomyelin (SM). Recombinant annexin A6 preferentially bound PS and PI (Figure 5A). To further evaluate the Ca^2+^ dependency and kinetics of recombinant annexin A6 binding to PS, surface plasmon resonance (SPR) interaction assays were performed with PS (+PS) or without PS (–PS) over a range of Ca^2+^ concentrations (0, 26, 52, 104, 208, 417, 833, and 2500 μM). Recombinant annexin A6 binding to PS increased with increasing concentrations of Ca^2+^, with appreciable binding at 50 μM and higher (Figure 5B). Relatively minimal recombinant annexin A6 binding occurred in the absence of PS, except where it was detected at 2500 μM Ca^2+^, and virtually no binding was present below 200 μM Ca^2+^ (Figure 5B). These studies support the idea that PS is a likely substrate for annexin A6’s membrane binding interactions during cell membrane repair.

### Annexin A6 senses phosphoinositides during cap formation.

Biological membranes are composed of lipid microdomains that regulate cell signaling events and membrane trafficking (46, 47). Annexins bind phospholipids, including PS and phosphatidylinositol-4,5-bisphosphate (PIP2), in response to changes in Ca^2+^ levels (48). Additionally, PS and PIP2 have been implicated in cell fusion and membrane repair located at the site of damage (24, 49, 50). To determine the contribution of phosphoinositides during membrane repair, we incubated myofibers in wortmannin, a known inhibitor of phosphatidylinositol-3-kinase. When used at higher concentrations (20 μM), wortmannin also inhibits phosphatidylinositol-4-kinase, leading to phosphatidylinositol 4-monophosphate and PIP2 depletion (51–53). Depletion of PIP2 after wortmannin treatment was verified through electroporation of the PIP2 fluorescent biosensor, PLC-PH-EGFP, in wild-type myofibers, which displayed a visible reduction in PLC-PH-EGFP signal with treatment (Figure 5C) (54). Myofibers from *Anxa6gfp* mice were subsequently treated with 20 μM wortmannin and subjected to laser-induced injury. Genomically encoded annexin A6GFP cap size was significantly reduced (3.3-fold; *P* < 0.002) by wortmannin treatment (Figure 5C). Combined, these results illustrate the dependency of annexin A6 repair cap formation on membrane lipid composition.

### Recombinant annexin A6 associates with injured myoblasts and myofibers.

**Figure 6:**
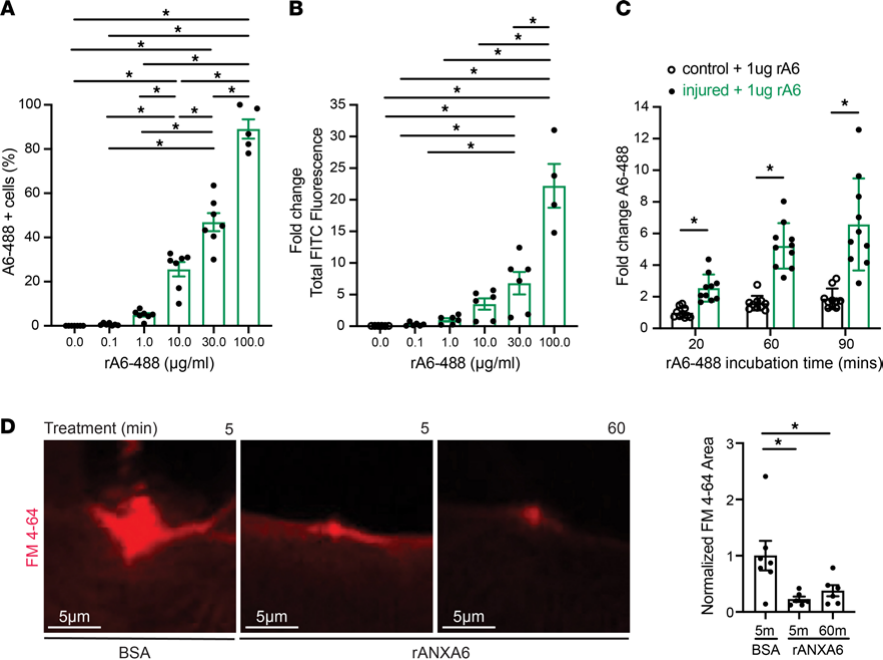
Recombinant annexin A6 associates with injured myoblasts and myofibers. (**A**) Rat L6 myoblasts were injured with LLO and then incubated with recombinant annexin A6 conjugated to 488 (rA6-488). The percentage of rA6-488–positive cells increased with increasing concentrations of annexin protein. (**B**) Total fluorescence intensity of rA6-488–positive cells increased with increasing concentrations of rA6-488, normalized to 1.0 μg/mL. (**C**) Increasing rA6-488 incubation time increased fluorescence signal of injured cells but not noninjured control cells. (**D**) Incubation of myofibers in recombinant annexin A6 (rANXA6) for either 5 or 60 minutes both reduced FM 4-64 dye uptake after injury compared with BSA-treated control myofibers. Scale bar: 5 μm. *n* ≥ 4 cell platings. *n* ≥ 6 myofibers from *n* = 4 isolations. *P < 0.05 by 1-way ANOVA.

Nygård Skalman and colleagues overexpressed annexin A6 from a plasmid in HeLa cells and found that it localized to the site of listeriolysin O–induced (LLO-induced) membrane injury (55). We developed a quantitative, cell-based assay that combined LLO-induced injury and flow cytometry using rat L6 myoblasts to evaluate the performance of recombinant A6 in an alternative injury paradigm. At the amino acid level, rat annexin A6 protein is 94.6% similar to human annexin A6 and 98.3% similar to mouse annexin A6 (23). L6 myoblasts were injured with LLO and then incubated with increasing concentrations of Alexa Fluor 488–labeled recombinant annexin A6 protein (referred to as rA6-488) in concentrations ranging from 0 to 100 μg/mL, and fluorescence as a surrogate measure of binding was quantified by flow cytometry. The percentage of rA6-488–positive cells increased with increasing concentrations of rA6-488, with nearly 100% of injured cells showing rA6-488 fluorescence at 100 μg/mL (Figure 6A). As another measure of binding, the total fluorescence intensity emitted by rA6-488 binding increased with increasing concentrations of protein, with levels 22-fold higher at 100 μg/mL than at 1 μg/mL (Figure 6B). Furthermore, when injured L6 myoblasts were incubated with the same concentration of rA6-488 but incubation time varied (20–90 minutes), fluorescence increased with increasing time (Figure 6C). To determine if timing of recombinant annexin A6 treatment altered function, we pretreated myofibers with recombinant annexin A6 for 5 minutes or 60 minutes and then subjected them to laser injury in the presence of FM 4-64 dye. Endpoint FM 4-64 dye uptake was similarly reduced with both 5 minutes and 60 minutes of pretreatment compared with controls treated with BSA (Figure 6D). Quantitation of FM 4-64 dye over time showed that FM 4-64 was significantly reduced with recombinant annexin A6 treatment throughout the imaging series (Supplemental Figure 3, A and B). These data indicate that recombinant annexin A6 binds disrupted myoblast and myofiber membranes and protects against membrane injury.

### Recombinant annexin localizes to the repair cap and enhances repair.

**Figure 7:**
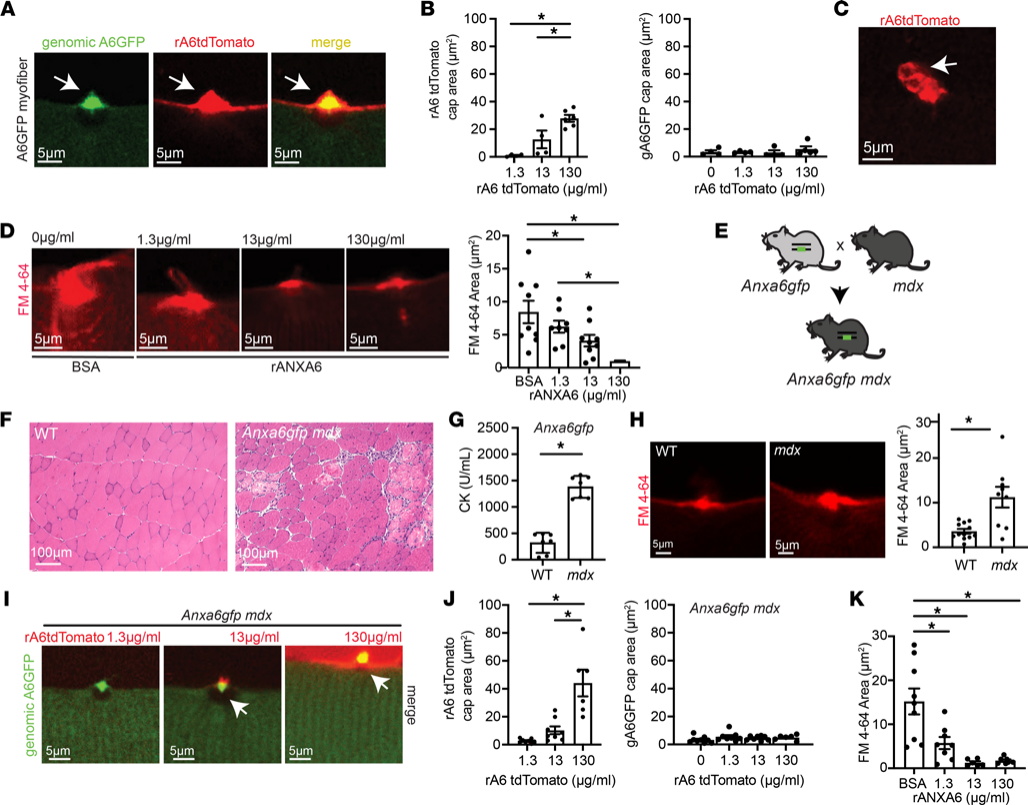
Recombinant annexin A6 cap size increases in a dose-dependent fashion, correlating with improved repair capacity. (**A**) Myofibers were isolated from *Anxa6gfp* mice and laser-damaged in the presence of rA6-tdTomato. rA6-tdTomato (shown in red) colocalized with genomically encoded annexin A6GFP (green) at the site of muscle membrane injury (white arrow). (**B**) rA6-tdTomato cap size increased with increasing concentrations of rA6-tdTomato, 1.3–130 μg/mL. Genomically encoded annexin A6GFP cap size did not change with increasing concentrations of rA6-tdTomato. (**C**) rA6-tdTomato formed membranous blebs at the site of membrane injury. (**D**) Dose-dependent reduction of FM 4-64 dye (red) uptake, a marker of membrane injury, with increasing concentrations of recombinant annexin A6. (**E**) *Anxa6gfp* mice were crossed with *mdx* mice to generate *mdx* mice expressing genomically encoded annexin A6GFP. (**F**) Dystrophic histopathology is present in *Anxa6gfp* *mdx* muscle. Scale bar: 100 μm. (**G**) Serum creatine kinase (CK) was elevated in *Anxa6gfp* *mdx* mice compared to *Anxa6gfp* controls (*n* = 7). (**H**) Increased FM 4-64 dye (red) in injured *Anxa6gfp* *mdx* myofibers compared with *Anxa6gfp* controls. (**I** and **J**) In *Anxa6gfp mdx* myofibers, genomically encoded annexin A6GFP formed a repair cap at the site of membrane injury. rA6-tdTomato cap size increased with increasing concentrations of rA6-tdTomato, 1.3–130 μg/mL. Genomically encoded annexin A6GFP cap size did not change significantly with varying concentrations of rA6-tdTomato in *Anxa6gfp mdx* myofibers. (**K**) Increasing concentrations of recombinant annexin A6 resulted in a dose-dependent reduction of FM 4-64 dye (red) uptake in dystrophic myofibers. Scale bar: 5 μm. A total of 4–9 myofibers from *n* ≥ 4 isolations. *P < 0.05 by 1-way ANOVA.

The above data demonstrate that annexin A6 expressed from the endogenous locus is recruited from its position within fibers to the plasma membrane to participate in resealing wounds. We evaluated whether exogenously added recombinant annexin A6 interacted with endogenously encoded annexin A6. To do this, we laser-injured *Anxa6gfp* myofibers in the presence of recombinant annexin A6 labeled with a carboxy terminal tdTomato fused tag (referred to as rA6-tdTomato). rA6-tdTomato localized to the site of membrane injury, colocalizing with genomically encoded annexin A6GFP (Figure 7A and Supplemental Video 4). rA6-tdTomato cap area increased with increasing concentrations (1.3–130 μg/mL) of available rA6-tdTomato protein (Figure 7B). However, the presence of rA6-tdTomato did not significantly increase genomically encoded annexin A6GFP cap size (Figure 7B). High-resolution imaging at the site of muscle membrane injury revealed the presence of rA6-tdTomato–containing membranous blebs (Figure 7C and Supplemental Video 5). To determine if the protective effect of rA6 was concentration dependent, we incubated myofibers in a range of recombinant annexin A6 protein concentrations (0–130 μg/mL) or BSA control. Subsequently, myofibers were incubated in FM 4-64 dye and subjected to laser injury. Myofibers pretreated with recombinant annexin A6 had a dose-dependent reduction in FM dye uptake, compared with control myofibers (Figure 7D), indicating higher levels of protection with increased concentrations of recombinant annexin A6 protein. These data demonstrate a role for annexin A6 in the immediate repair response required to seal membrane lesions independent of overexpression systems.

We next assessed the role of annexin A6 in dystrophic muscle, which continually undergoes bouts of injury and repair. *Anxa6gfp* mice were crossed with the *mdx* model of Duchenne muscular dystrophy to generate *Anxa6gfp mdx* mice (Figure 7E). The *mdx* mouse model lacks dystrophin expression, resulting in a fragile sarcolemma that is prone to injury (56, 57). *Anxa6gfp mdx* mice displayed typical features of muscular dystrophy (Figure 7F). Serum creatine kinase (CK), a clinically relevant biomarker of injury, was significantly elevated in the serum of *Anxa6gfp mdx* mice compared with healthy *Anxa6gfp* mice (Figure 7G). *Anxa6gfp* *mdx* myofibers were isolated and laser-injured. The amount of FM 4-64 after injury was significantly elevated in *Anxa6gfp mdx* myofibers compared with *Anxa6gfp* myofibers, consistent with the known fragility of dystrophin-deficient muscle (Figure 7H). Additionally, *Anxa6gfp mdx* myofibers were isolated and laser-injured in the presence of rA6-tdTomato. Similar to WT muscle, annexin A6GFP localized in a mainly sarcomeric pattern prior to injury and then formed a repair cap at the membrane lesion after laser-wounding (Figure 2E and Figure 7I). The fragile nature of dystrophin-deficient myofibers makes it challenging to isolate intact fibers for the laser assay, as fibers injured during isolation cannot be used in the assay. Hence, comparisons between healthy and dystrophic myofibers should take this into account and may underestimate the degree of injury in dystrophic fibers. A6-tdTomato localized to the site of dystrophic membrane injury, colocalizing with genomically encoded annexin A6GFP at the lesion (Figure 7, I and J). Genomically encoded annexin A6GFP cap size in *mdx* myofibers was not significantly altered by the presence of rA6-tdTomato (Figure 7, I and J). However, pretreatment with recombinant annexin A6 had a dose-dependent effect on myofiber FM dye uptake, reducing dye influx compared with control-treated myofibers (Figure 7K). Together, these findings show that recombinant annexin A6 protein increases exogenous cap size with increased concentrations, correlating with increased repair capacity in both healthy and dystrophic muscle.

### Recombinant annexin A6 localizes to injured neurons.

**Figure 8:**
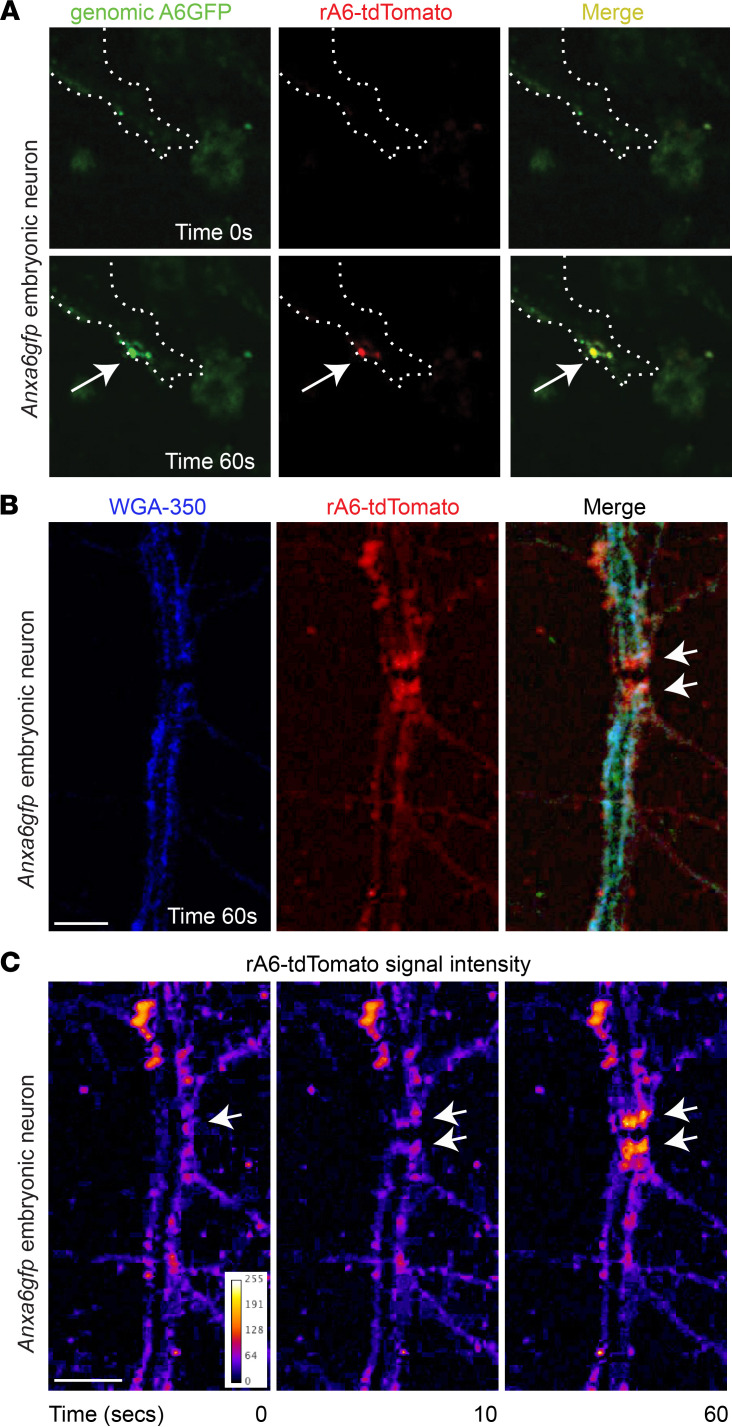
Recombinant annexin A6 binds neuronal membrane lesions. (**A**) Embryonic neurons were isolated from *Anxa6gfp* mice, matured, and laser damaged in the presence of rA6-tdTomato. rA6-tdTomato (shown in red) colocalizes with genomically encoded annexin A6GFP (green) at the site of muscle membrane injury (white arrow). Neuron outlined in white dotted line. (**B**) After transection of *Anxa6gfp* neuronal processes, rA6-tdTomato (red) localizes at the stumps of the severed process (white arrows). WGA-350 (blue) outlines the neuron. (**C**) rA6-tdTomato fluorescence signal increases at the process stumps with time (white arrows). Multiple neurons from *n* ≥ 3 mice. Scale bar: 5 μm.

The process of neuron regeneration after axonal crush or severing requires resealing of the membrane prior to growth cone formation and regeneration (58). To evaluate the binding potential of recombinant annexin A6 to injured neurons, embryonic neurons were isolated and laser-injured in the presence of rA6-tdTomato. In the first injury assay, the neuronal membrane was nicked with the laser, similar to the protocol used for skeletal myofibers. With this type of injury, rA6-tdTomato localized to the site of neuron membrane injury, colocalizing with genomically encoded annexin A6GFP at the repair cap (Figure 8A). In the second injury assay, the laser was used to fully transect the neuronal process, creating 2 stumps at the injury site. Wheat germ agglutinin (WGA) was used to label the neuron membrane, which showed clear disruption of the neuronal process after transection (Figure 8B). Four seconds after transection, when the first image was acquired, rA6-tdTomato was detectable at the transected stumps (Figure 8, B and C, and Supplemental Video 6). rA6-tdTomato fluorescence intensity at the severed stumps continued to increase through the 60 seconds of imaging (Figure 8C). Therefore, recombinant annexin A6 binds disrupted neuronal membranes after the smaller nicking injury and after full transection.

## Discussion

### Impact of annexin A6 expression level on membrane repair.

Prior studies examining the role of annexin A6 in membrane repair relied on plasmid-mediated overexpression, which could alter the kinetics and efficiency of membrane repair. In this study, we relied on a genomically encoded annexin A6GFP, expressed at lower levels than normal annexin A6 protein, to document the role of annexin A6 in repair cap formation. This decrease in in vivo expression of total annexin A6 did not appear to have deleterious effects on muscle histology, and repair complex formation remained readily visible. A recent study using shRNA to reduce annexin A6 expression in cultured human muscle cells (59) reported a reduction in repair capacity after acute reduction in annexin A6, which appeared to correlate with degree of annexin A6 reduction. In vivo, it is possible that there is compensation by other proteins, including other annexins, to offset the reduction in annexin A6 in *Anxa6gfp* mice. However, in the previously generated *Anxa6*-null mice, increased expression of annexin A1, A2, or A5 protein was not detected in skeletal muscle, heart, or liver (44). These data combined with our findings suggest that the small amount of functional annexin A6 is sufficient to promote repair, consistent with annexin A6 being a modifier gene and not a primary disease-causing gene (30).

### Recombinant annexins mediate closure of membrane lesions.

**Figure 9:**
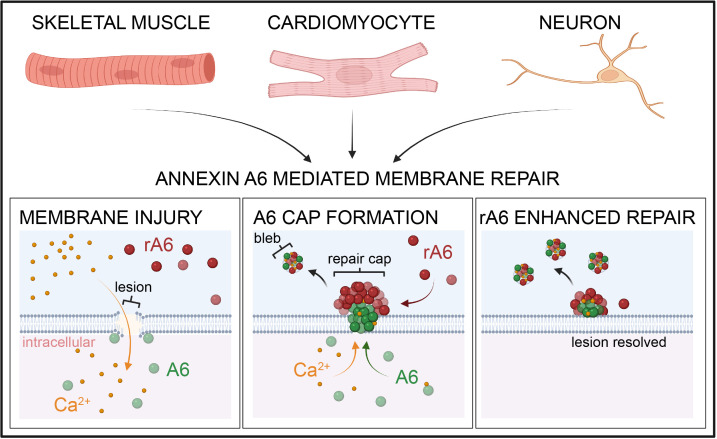
Model of annexin A6–mediated membrane repair in skeletal muscle, cardiomyocytes, and neurons. Upon plasma membrane breach, extracellular Ca^2+^ enters the damaged cell. Annexin A6 (A6) binds Ca^2+^, translocates to the site of membrane injury targeting exposed phospholipids such as PS, and forms a repair cap at the lesion. Extracellular recombinant annexin A6 (rA6) localizes to the repair cap at the site of injury, enhancing repair capacity.

Using data mainly derived from artificial membranes and recombinant purified proteins, annexin A6 has been implicated in membrane bending and the constriction forces needed to pull wound edges together for eventual fusion (60, 61). Our studies provide in vivo support for a role for annexin A6 in wound closure because we observed annexin A6GFP–positive membrane blebs emanating from the annexin repair cap and exogenously added recombinant annexin A6 protein at these same sites. Together, these data support that annexin A6 protein binds exposed membrane phospholipids at the site of injury from the cell interior or exterior (model in Figure 9). Boye et al. also demonstrated that annexins A1 and A2 remodel artificial membranes, promoting rolling and blebbing (60, 61). Although recombinant annexin A6 protein elicits a substantial, dose-dependent improvement in repair, it is possible that an annexin cocktail containing multiple annexin proteins may provide additional benefit. The data presented in Figure 6 support a model where recombinant annexin A6 exponentially binds to injured membranes. Annexin A6 has many known binding partners, including itself (62). Annexin A4, another annexin family member, self-associates at the plasma membrane in a Ca^2+^-dependent fashion, reducing mobility of other membrane-associated proteins (63, 64). Regulating the mobility of membrane-associated proteins at the site of injury may be a common function of annexin family members aiding in plasma membrane preservation during the repair process.

### Annexin A6 in cardiac injury and disease.

Cardiomyocytes are a terminally differentiated cell type with limited ability to regenerate. Therefore, cell survival is crucial for preserving cardiac function. Membrane repair is vital for cardiac membrane stability, and impairments in membrane repair may lead to heart disease and failure (reviewed in 65). The role of annexin A6 in cardiomyocyte repair is understudied and has primarily relied on expression and localization analyses. Annexin A6 is expressed in the healthy heart at levels higher than annexin A2 and A5 (66, 67). In a study of human idiopathic dilated cardiomyopathy (DCM), left ventricle samples showed a 1.2-fold increase in annexin A6 protein expression in failing hearts (*n* = 11) compared with nonfailing hearts (*n* = 9), with annexin A6 localization at the sarcolemma and transverse tubules (66). Conversely, Song et al. found that the expression of annexin A6 was markedly reduced at the mRNA and protein level in end-stage human hearts failing due to coronary artery disease (*n* = 6) or idiopathic DCM (*n* = 6) compared with controls (*n* = 6) (67). Direct comparison of these studies is limited by differential protein extraction methods and the increasing appreciation that idiopathic DCM is not a single disease entity. In a porcine hypertension and left ventricle hypertrophy model of heart failure, annexin A6 protein levels were upregulated in the failing heart, with annexin A6 localizing to the sarcolemma (68). The observation that annexin A6 localizes to the site of cardiomyocyte injury, forming a repair cap at the membrane lesion, supports a role for annexin A6 in heart disease associated with increased cellular breakdown.

### Annexins A6 in neuronal injury.

Similar to muscle, neuronal membrane damage can occur from physical trauma, from degenerative processes, or as a secondary consequence of a primary disease. Unrepaired damage in neurons leads to cell degeneration and death, with devasting physical consequences. As in skeletal muscle, an increase in intracellular Ca^2+^ occurs when the membrane is breached. A persistent rise in intracellular Ca^2+^ in neurons may lead to dysregulated ion gradients, protease activation, mitochondrial dysfunction, and apoptosis (69). Therefore, timely repair of lesions in neuronal membranes is crucial. Previous studies have shown using plasmid overexpression that annexin A1, A2, A4, A5, and A6 assemble at the plasma membrane of injured neuroblastoma cells in vitro (43). Our results expand on these findings, demonstrating that genomically encoded annexin A6 was expressed in maturing primary neurons at levels sufficient to form a repair cap at the site of injury. The immediate localization of annexin A6 into a repair cap in neurons is similar to that in muscle and suggests annexin A6 orchestrates repair of neuronal membranes. The role of annexin A6 in facilitating neuronal repair is further supported by the observation that externally delivered recombinant annexin A6 bound to the damaged area on the neuronal plasma membrane both after generation of a small lesion and after transection. Studies are ongoing to determine the therapeutic potential of recombinant annexin A6 to enhance neuronal repair and/or prevent neuronal damage.

In summary, this work demonstrates that annexin A6 forms repair caps that mediate resealing, and this process is conserved across multiple cell types. Importantly, we show that exogenously administered recombinant annexin A6 rapidly binds to the damaged membrane in skeletal muscle, cardiomyocytes, and neurons, suggesting a broad role for annexin A6 in repair.

## Methods

### Animals.

Wild-type mice from the 129T2/SvEmsJ background were bred and housed in a specific pathogen–free facility on a 12-hour light/12-hour dark cycle and fed ad libitum. *129T2/SvEmsJ* (129T2) mice were originally purchased from The Jackson Laboratory (stock 002065). Two- to three-month-old male and female mice were used for all experiments. mdxC57BL10 mice were obtained from The Jackson Laboratory (stock 001801).

### Generation of gene-edited mice.

The *Anxa6* TurboGFP mouse line was generated by the Northwestern University Transgenic & Targeted Mutagenesis Core Facility. CRISPR/Cas9 technology was used to insert the TurboGFP coding sequence before the *Anxa6* stop codon, creating a fusion construct. Briefly, 2 guide RNAs (gRNAs) were identified using Broad Institute online software (https://portals.broadinstitute.org/gpp/public/analysis-tools/sgrna-design). The gRNA sequences are gRNA6, 5*′*-GCTTGCTCTGTGTGGCGGAG-3*′*, and gRNA10, 5*′*-AGAGGGGGCCCTCTGAGGTC-3*′*. Each gRNA was engineered into the PX459 V 2.0 Cas9 vector (Addgene 62988) as previously described (70). A repair vector was synthesized, then engineered into the pUC57 backbone by GeneWiz. This vector encodes TurboGFP flanked by 700 bp homology arms. Three silent mutations were introduced in *Anxa6* to destroy the gRNA6 recognition site.

Both gRNA/Cas9 plasmids (0.5 μg/each) and the repair template (2 μg) were introduced into 129S6 embryonic stem (ES) cells via nucleofection (Nucleofector 2b, Lonza). After 24 hours, ES cells were subjected to puromycin selection for 48 hours. ES cell clones were isolated and genotyped for insertion of the repair template into the *Anxa6* locus. Targeted clones were microinjected into blastocyst stage C57BL/6J (strain 000664) embryos, which were then surgically transferred into the reproductive tract of recipient females. Chimeric mice were genotyped for the Anxa6 TurboGFP allele.

### PCR and genomic DNA analysis.

Genomic DNA was isolated from mouse tail tissues. Gene-edited mice were genotyped based on the presence of the wild-type annexin A6 and/or TurboGFP. PCR was performed using the following primer sequences: (a) forward primer: 5*′* CTAGGCCGATGGCTGCTA 3*′*, (b) reverse primer for wild-type annexin A6: 5*′* CAATGGCTTGGTCAGGTCAC 3*′*, and (c) reverse primer for tGFP: 5*′* ACTTCTCGATGCGGGTGTTGGTG 3*′*. Products were amplified by PCR using Phusion High-Fidelity DNA Polymerase (New England Biolabs) with the following cycle conditions: initial denaturation 98°C, 45 seconds followed by 98°C, 10 seconds; 64°C, 30 seconds; 72°C 30 seconds for 35 cycles, and a final extension 72°C for 5 minutes. Products were run on 2% agarose gels with ethidium bromide. Additionally, Sanger sequencing was performed on the amplified product to verify in-frame GFP insertion. 129/S6 (SvEvTac) ES cell genomic DNA was isolated and sequenced to confirm the absence of the annexin A6 truncated polymorphism.

### Plasmids.

A plasmid encoding annexin A6 with a carboxy terminal TurboGFP tag was obtained from Origene. Subcloning of annexin A6 to replace the GFP tag with tdTomato (Addgene) was performed by Mutagenix. Constructs were sequenced to verify mutagenesis. Plasmid DNA was isolated using the QIAGEN EndoFree Plasmid Maxi Kit.

### Sequence comparison and schematics.

Snapgene and Lasergene were used to view and align chromatograms.

### Protein isolation.

Muscles were dissected and flash-frozen. Tissues were lysed in whole tissue lysis buffer (50 mM HEPES pH 7.5, 150 mM NaCl, 2 mM EDTA, 10 mM NaF, 10 mM Na-pyrophosphate, 10% glycerol, 1% Triton X-100, 1 mM phenyl-methylsulfonyl fluoride [PMSF], 1× cOmplete Protease Inhibitor Cocktail [catalog 11697498001 CO-RO; Roche]) and homogenized using a bead beater tissue homogenizer (BioSpec).

### Immunoblotting.

The protein concentration of the muscle or cell lysate was determined using the Quick Start Bradford Protein Assay (catalog 5000205; Bio-Rad Laboratories). Proteins were heated to 70°C in 2× Laemmli buffer and were separated on 7.5% Mini-PROTEAN TGX Precast Protein Gels, 15-well, 15 μL (catalog 4561026; Bio-Rad Laboratories) and transferred to Immun-Blot PVDF Membranes for Protein Blotting (catalog 1620177; Bio-Rad Laboratories). Blocking and antibody incubations were done using StartingBlock T20 (TBS) Blocking Buffer (catalog 37543; Thermo Fisher Scientific). Primary antibodies used were annexin A6 (catalog 31026; Abcam) and TurboGFP (Evrogen, catalog AB513) used at 1:1000 diluted in starting block. Secondary antibodies conjugated to horseradish peroxidase were used at 1:5000 (catalog 111-035-003; Jackson ImmunoResearch Laboratories). SuperSignal West Pico Chemiluminescent Substrate and SuperSignal West Femto Maximum Sensitivity Substrate (catalog 34080 and 34096; Thermo Fisher Scientific) were applied to membranes, and membranes were visualized using an Invitrogen iBright CL1000 Imaging System (catalog A32749; Thermo Fisher Scientific). Pierce Reversible Protein Stain Kit for PVDF Membranes (including MemCode; catalog 24585; Thermo Fisher Scientific) was used to stain the entire blot to ensure complete transfer and equal loading. Immunoblot bands were quantified using FIJI gel analysis tools (NIH).

### Membrane lipid assay.

Lipid strip assays were performed per manufacturer’s instructions (Echelon Biosciences P-6003-2). Briefly, membrane was blocked with 5 mL TBS with 0.25% Triton X-100 (TBS-T) + 3% BSA for 1 hour at room temperature. Protein (1 μg/mL recombinant annexin A6 in TBS-T + 3% BSA + 1 mM calcium) was incubated on the membrane for 1 hour at room temperature. Membranes were rinsed 3 times for 5 minutes in TBS-T and then incubated with anti–HIS-HRP (Thermo Fisher Scientific MA1-21315-HRP) diluted 1:500 in TBS-T + 3% BSA for 1 hour at room temperature. Membranes were rinsed 3 times for 5 minutes in TBS-T and developed with 2 mL TMB Precipitating (Echelon Biosciences K-TMBP) for 2–20 minutes.

### Electroporation and myofiber isolation.

Flexor digitorum brevis fibers were transfected with endo-free plasmid DNA by in vivo electroporation. Methods were described previously (23, 24, 71, 72). Briefly, fibers were dissociated in 0.2% BSA plus collagenase type II (catalog 17101, Thermo Fisher Scientific) for 90–120 minutes at 37°C in 10% CO_2_. Fibers were then moved to Ringers solution and placed on MatTek confocal microscopy dishes (catalog P35G-1.5-14-C).

### Cardiomyocyte isolation.

Mice were treated with 50 U heparin intraperitoneally 20 minutes before sacrifice. Mice were anesthetized under 5% vaporized isoflurane mixed with 100% oxygen. A thoracotomy was performed, and the heart and lungs were rapidly excised and submerged into ice-cold Tyrode solution without calcium (143 mM NaCl, 2.5 mM KCl, 16 mM MgCl_2_, 11 mM glucose, and 25 mM NaHCO_3_, pH adjusted to 7.4). The ascending aorta was dissected out of the surrounding tissue and cannulated with an animal feeding needle (7900, Cadence Science) and secured with a 6-0 silk suture. The heart was initially perfused with 1 mL of ice-cold calcium-free Tyrode solution before being transferred to a Langendorff apparatus (Radnoti). Hearts were perfused with 37°C calcium-free Tyrode solution using a constant pressure (65 cm vertical distance between the buffer reservoir and cannula tip) for 1 to 2 minutes before perfusion for 5.5 minutes with digestion solution (0.15% collagenase type 2 [Worthington Biochemical], 0.1% 2,3-butanedione monoxime (MilliporeSigma), 0.1% glucose, 100 U/mL penicillin/streptomycin, 112 mM NaCl, 4.7 mM KCl, 0.6 mM KH_2_PO_4_, 40 μM CaCl_2_, 0.6 mM Na_2_HPO_4_, 1.2 mM MgSO_4_, 30 μM phenol red, 21.4 mM NaHCO_3_, 10 mM HEPES, and 30 mM taurine, pH adjusted to 7.4). The heart was removed from the cannula, triturated with a transfer pipette, and filtered through a 100 μm cell strainer (Thermo Fisher Scientific). Cardiomyocytes were allowed to pellet by gravity for 7 minutes, followed by aspiration of digestion media and washing with stop buffer (formulated identically to digestion solution except with no collagenase and with 1% BSA). Cells were again allowed to gravity pellet, followed by a wash in stop buffer without BSA. Cardiomyocytes were tolerated to calcium by adding Tyrode buffer with 0.3 mM CaCl_2_ dropwise. Cell culture dishes were coated with 20 μg/mL laminin (catalog 23017-015; Gibco, Thermo Fisher Scientific) for 1 hour at room temperature. Laminin solution was aspirated followed by plating of cardiomyocytes for 1 hour to allow cell adhesion before experimentation.

### Multiphoton laser injury and imaging.

Isolated fibers were subjected to laser-induced damage at room temperature using the Nikon A1R MP+ multiphoton microscope as described previously (23). Imaging was performed using a 25 × 1.1 NA objective directed by the NIS-Elements AR imaging software. GFP and FM 4-64 FM 4-64 (catalog T13320; Thermo Fisher Scientific) were excited using a 920 nm wavelength laser, and emission wavelengths of 575 nm and 629 nm, respectively, were collected. To induce laser damage on isolated myofibers, a diffraction-limited spot (diameter approximately 410 nm) was created on the lateral membrane of the myofiber using a 920 nm wavelength laser at 10%–15% laser power for 1 second. Time-lapse images were collected as follows: 1 image was collected prior to damage, 1 image upon damage, then every 8 seconds for 80 seconds (10 images) followed by every 30 seconds for 5 minutes (10 images). At the end of the time-lapse image series, *Z*-stack images were collected at 250 nm intervals through the damaged site on the myofiber directed by the NIS-Elements AR imaging software. Fluorescence intensity and cap area were measured using FIJI. To damage cardiomyocytes, the cells were isolated and plated on laminin-coated MatTek confocal microscopy dishes as described above. The cells were incubated for 1 hour at 37°C to allow cell attachment. Prior to laser damage, the cells were incubated in Tyrode buffer containing 0.5 mM CaCl_2_ and damaged as described. To damage neurons, the cells were grown in 35 mm culture dishes in growth media. Prior to damage, the cells were washed twice in PBS and incubated in Ringer’s buffer containing 1 mM CaCl_2_ and damaged as described above.

For recombinant protein studies, myofibers were isolated from mice as described above. Myofibers were incubated in specified concentrations of recombinant annexin A6 and 1 mM Ca^2+^ Ringers or BSA control. Cap size was assessed from acquired images in FIJI. FM 4-64 (2.5 μm) was added to the myofibers just prior to imaging. Images were acquired and quantitated as described above, and FM 4-64 was quantified as described (23, 24). FM 4-64 fluorescence area at imaging endpoint was quantified by outlining the FM 4-64 accumulation at the injury site using the “measure” tool in FIJI. FM 4-64 area and fluorescence over time were quantified by measuring the FM area and fluorescence intensity at the injury site within each frame. F/F0 was calculated as FM fluorescence intensity divided by the intensity in frame 0. Isolated myofibers were treated with 20 μM wortmannin (catalog 12-338; MilliporeSigma).

### Immunostaining and immunofluorescence imaging.

Extensor digitorum longus muscle was harvested and fixed in 4% paraformaldehyde. Fibers were rinsed and blocked with starting block with 0.1% Triton X-100 for 1 hour. Fibers were incubated overnight at 4°C with anti–annexin A6 antibody (catalog LS-B7015-50, LSBio; 1:100). Fibers were subsequently incubated with donkey anti-rabbit Alexa Fluor 488 (Thermo Fisher Scientific; 1:2500). Fibers were mounted in ProLong Gold Antifade (catalog P36930, Thermo Fisher Scientific) and images acquired on a Keyence BZ-X800 microscope with a 100× objective. All image acquisition settings were the same among genotypes.

### Histology.

Hematoxylin and eosin staining was performed on isolated muscle per manufacturer’s protocol. Sections were imaged on a Keyence BZ-X800 microscope with a 20× objective.

### Serum CK.

Serum CK was analyzed using the EnzyChrome creatine kinase assay kit (ECPK-100; BioAssay Systems) following the manufacturer’s protocol. Samples were measured in duplicate and averaged. Results were measured with the Synergy HTX multimode plate reader (BioTek).

### Neuron isolation and immunoblot.

Mixed cortical and hippocampal neurons were isolated from day 15.5–16.5 annexin A6GFP or C57BL/6J mouse embryos via dissociation at 37°C in 0.25% trypsin. Neurons were plated in poly-l-lysine–coated 12-well plates (750,000 cells per well) or MatTek glass-bottomed, 3 cm dishes (450,000 cells per dish) containing neurobasal media supplemented with 2% B-27, 500 μM glutamine, 10% horse serum, and 2.5 μM glutamate. After 2 hours, the media were replaced with neurobasal media with 2% B-27 and 500 μM glutamine. All cell culture reagents were from Thermo Fisher Scientific. For immunoblotting, cells in 12-well plates were lysed in in RIPA buffer (150 mM NaCl, 1% IGEPAL CA-630, 0.5% sodium deoxycholate, 0.1% SDS, 50 mM Tris pH 8, 1 mM PMSF) with Protease Inhibitor Cocktail III (Calbiochem) and Halt Phosphatase Inhibitor Cocktail (Thermo Fisher Scientific). Lysates were centrifuged at 10,000 rpm, 4°C, 10 minutes, and the supernatant protein was quantified by BCA (Thermo Fisher Scientific). A total of 10 μg protein was separated on Invitrogen NuPAGE Bolt 4 to 12% Bis-Tris gels and transferred overnight to PVDF (MilliporeSigma). Membranes were blocked in Thermo Fisher Scientific SuperBlock and then probed for 1 hour at room temperature with either anti–TurboGFP (1:1000) in 10% SuperBlock in TBS 0.1% Triton X-100, or anti-GAPDH (Cell Signaling Technology 14C10) in 5% milk in TBS 0.1% Triton X-100 followed by horseradish peroxidase–conjugated anti-rabbit antibody (Vector Laboratories PI-1000, 1:5000). Blots were visualized using the Pierce reagents West Femto (TurboGFP) or SuperSignal West Pico (GAPDH), and signals were imaged using a FluorChem imager (ProteinSimple) and then quantified with AlphaView software (ProteinSimple). TurboGFP signal was normalized to GAPDH, and 2-tailed *t* test was done using InStat software (GraphPad Software, Inc.)

### Brain sectioning and imaging.

Two- to four-month-old heterozygous annexin A6GFP or wild-type mice were euthanized and transcardially perfused with ice-cold PBS containing protease and phosphatase inhibitors. After perfusion, the brain was bisected, and 1 hemibrain was drop-fixed in 4% paraformaldehyde/PBS and cryopreserved in 30% w/v sucrose/PBS for sectioning. The other hemibrain was flash-frozen in liquid nitrogen for biochemical analysis. A total of 30 μm coronal floating brain sections were cut and stained as follows. Sections were washed 3 times in TBS, incubated in 16 mM glycine in TBS-T, and blocked first in 5% donkey serum in TBS-T, then in 1% BSA in TBS-T. Sections were incubated overnight at 4°C with anti-TurboGFP (1:500) and mouse anti-NeuN (MilliporeSigma, MAB377, 1:1000) in 1% BSA TBS-T. The following day, they were incubated with 1:750 donkey anti-rabbit Alexa Fluor 488 (Thermo Fisher Scientific catalog A32790) and donkey anti-mouse Alexa Fluor 594 **(**Thermo Fisher Scientific catalog A32744). All staining was performed at the same time. Sections were mounted with ProLong Gold Antifade (Thermo Fisher Scientific catalog P36930) and images acquired on a Nikon A1R or W1 confocal microscope with a 20× or 40× objective, using NIS-Elements software. All image acquisition settings were the same among genotypes.

### Recombinant protein production.

Recombinant annexin A6 protein and annexin A6-tdTomato protein were generated by Evotec using *E*. *coli* and Expi293 cells and standard methods. Media were purified using immobilized metal affinity chromatography chromatography. The final recovery of purified recombinant annexin A6 protein was diluted in TBS with an endotoxin level at approximately 1.5 EU/mg, with a purity more than 80%. Recombinant annexin A6 was labeled with Alexa Fluor 488 using standard methods (catalog A10235, Thermo Fisher Scientific).

### In vitro injury and binding.

A stock of 50 ng/mL LLO (catalog ab83345; Abcam) in PBS without calcium and without magnesium (PBS–/–) was prepared on ice. L6 rat myoblasts (ATCC CRL-1458) were trypsinized and resuspended in PBS–/– to achieve a concentration of 10,000 cells/μL. A total of 1,000,000 cells were added to each tube of the prepared LLO and incubated on ice for 5 minutes. After 5 minutes, cells were pelleted, rinsed twice, and resuspended in PBS with 0.45 nm Ca^2+^ with varying concentrations of rA6-488 (0 to 100 μg/mL). Cells were incubated at 25°C for 5 minutes. A total of 2 μL of SYTOX Red dye (catalog S34859; Thermo Fisher Scientific) was added to each tube of cells and incubated at 25°C for an additional 10 minutes. Cells were rinsed and then resuspended in 300 μL of PBS–/–. Flow cytometry was performed on the BD Accuri C6 flow cytometer. A cell count of 30,000 was achieved for each tube. Analysis was performed using FlowJo software (BD).

### Liposome preparation.

The preparation of uniform ~100 nm diameter liposomes was carried out as described previously (73). PS, SM, cholesterol (CH), PE, and PC were commercially purchased (MilliporeSigma). PS was resuspended in chloroform/methanol solution to make a 10.7 mg/mL (27.77 mM) stock solution, PC in chloroform to make a 25 mg/mL stock (31.80 mM), PE in chloroform to make a 25 mg/mL stock (33.60 mM), CH in chloroform to make a 100 mg/mL stock (258.63 mM), and SM in methanol to make a 25 mg/mL stock (34.20 mM). Liposomes were prepared +PS and –PS. The composition ratios for liposome preparations are +PS (3.0 PC:1.5 PE:3.0 CH:1.5 SM:1.0 PS) and –PS (3.0 PC:1.5 PE:3.0 CH:1.5 SM). All lipids were at room temperature before preparing the +PS and –PS mixtures. Each lipid mixture was dried for 15–20 minutes under a steady and gentle stream of nitrogen. Each dried lipid mixture was resuspended in 1 mL of buffer (50 mM HEPES pH 7.3, 50 mM NaCl) to make a 10 mM of +PS and ~9 mM stock of –PS in glass vials. The glass vials were sealed with parafilm and sonicated for ~10 minutes. The liposome preparation was carried out using an Avanti Polar Lipids mini extruder (Croda International Plc, ref. 74). The lipid mixture was cycled through the extruder for 20–25 cycles. The liposome mixture was transferred to glass scintillation vials and stored at 2°C–8°C until use.

### SPR binding studies.

All SPR studies were performed on a Biacore 8K+ instrument (Cytiva) at 25°C. A series S L1 chip (lipophilic groups are covalently attached to carboxymethylated dextran, making the surface suitable for direct attachment of lipid membrane vesicles) (Cytiva) was used for the annexin A6/lipid interaction studies. Briefly, the L1 sensor chip was equilibrated in running buffer (10 mM HEPES, pH 7.4, 150 mM NaCl) and conditioned with two 30-second injections of 40 mM octyl glucoside at 10 μL/min before liposome immobilization (75). We captured 0.5 mM +PS and –PS liposomes (flow rate 2 μL/min) onto the active and reference flow cell surfaces, respectively, to approximately 10,000 RU to form the lipid bilayer. This was followed by two 60-second injections of 10 mM NaOH at 10 μL/min to remove any unbound liposomes on the L1 chip. Initially, the Ca^2+^ dependence of annexin A6 binding to +PS and –PS lipid was tested. The CaCl_2_ concentration in the running buffer was varied between 0 and 2.5 mM (0, 26, 52, 104, 208, 417, 833, or 2500 μM), and the binding signal RU and kinetics of the annexin A6/lipid interactions were analyzed. This was followed by dose-response kinetics studies on the annexin A6/+PS lipid interactions at 100 μM CaCl_2_. Since –PS showed very minimal binding at 100 μM CaCl_2_, it was used as a negative control lipid and captured on the reference flow cell surface. +PS was captured on the active flow cell surface. Parallel dose-response kinetics was run with 8 concentrations (0.78–100 nM) at 2-fold dilutions on 8 channels of the sensor chip. annexin A6 was injected at 50 μL/min with association time of 240 seconds and dissociation time of 600 seconds. We analyzed all data using Biacore Insight Evaluation software (ver. 3.0.12; Cytiva). Raw sensorgrams were reference-subtracted and blank-buffer-subtracted before kinetic and affinity analysis to account for nonspecific binding and injection artifacts. Association (*K_A_*/M/s) and dissociation (*K_D_*/s) rate constants and binding affinity (*K_D_*) values were determined using a 1:1 kinetics binding model. The closeness of fit between the experimental data and fitted curves was assessed using χ^2^ (average squared residual).

### Statistics.

Statistical analyses were performed with Prism (GraphPad Software, Inc.). Comparisons relied on ANOVA (1-way ANOVA for 1 variable, 2-way ANOVA for 2 variables). Otherwise, unpaired 2-tailed *t* tests were performed. *P* value less than or equal to 0.05 was considered significant. Data were presented as single values where appropriate. Error bars represent ± SEM.

### Study approval.

All procedures using mice followed the *Guide for the Care and Use of Laboratory Animals* (National Academies Press, 2011) and were approved by Northwestern University’s Institutional Animal Care and Use Committee.

## Author contributions

EB performed genomic amplification by PCR and prepared and imaged myofibers. PGTP performed histology. KSF and CCO performed quantitative PCR and fluorescence imaging. MH performed mouse husbandry. AK, KRS, RV, GC, and AT performed the neuron isolations, the neuron staining, and related immunoblots. LAV and NLR performed immunoblots and lipid binding studies. NLR performed LLO injury. PRBJ performed SPR binding and kinetics studies. LAV, ARD, and AML performed immunofluorescence and imaging. ARD and EMM designed the studies, analyzed the data, and wrote the manuscript.

## Supplementary Material

Supplemental data

Supplemental video 1

Supplemental video 2

Supplemental video 3

Supplemental video 4

Supplemental video 5

Supplemental video 6

## Version 1 07/22/2022

Electronic publication
