# Supplementary material for: A conserved annexin A6–mediated membrane repair mechanism in muscle, heart, and nerve
Source: JCI Insight. 2022 Jul 22;7(14):e158107. doi: 10.1172/jci.insight.158107 (PMC9431694; doi:10.1172/jci.insight.158107)
Supplement: Supplemental data [file jciinsight-7-158107-s056.pdf]

Supplemental Figures and Legends For "A conserved annexin A6-mediated membrane repair mechanism in muscle, heart, and nerve"

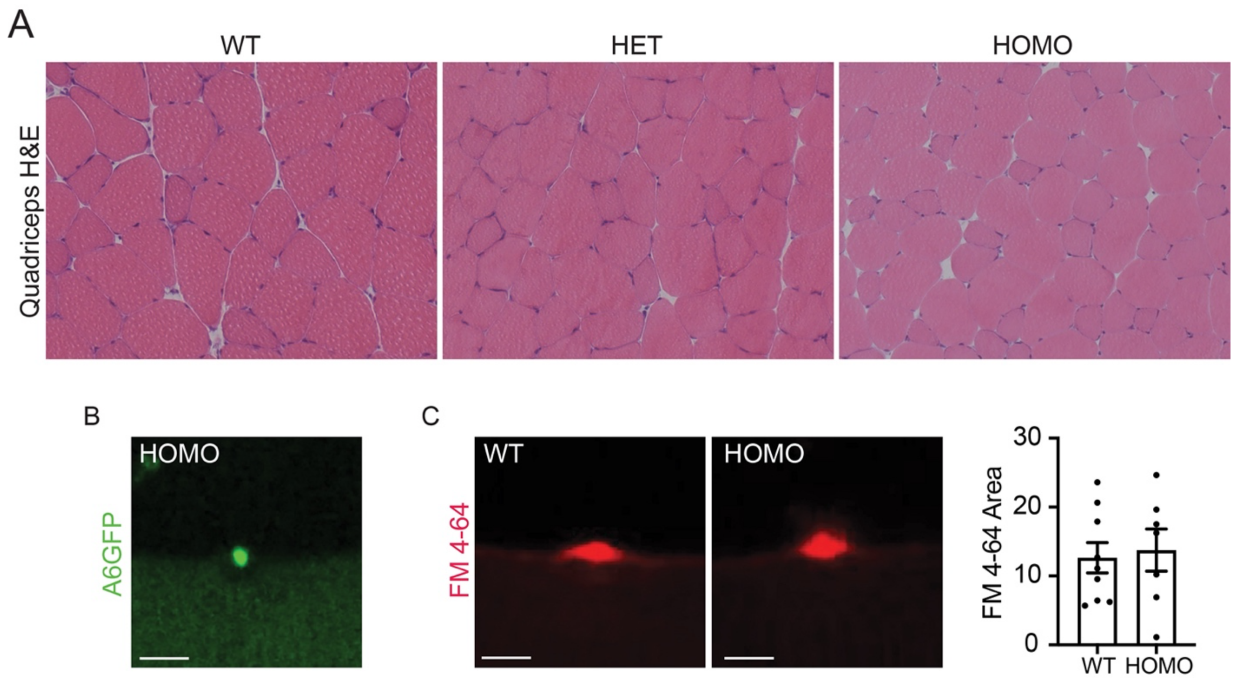

Supplemental Figure 1. A) Grossly normal muscle histopathology in heterozygous and homozygous *Anxa6gfp* tissue compared to wildtype control muscle. **B**) Upon laser-induced membrane injury, annexin A6GFP localized to the repair cap in homozygous *Anxa6gfp* myofibers. **C**) Similar FM 4-64 uptake after laser-induced membrane injury of wildtype and homozygous *Anxa6gfp* myofibers. Scale 5μm. n=3 mice per genotype, n ≥ 7 myofibers.

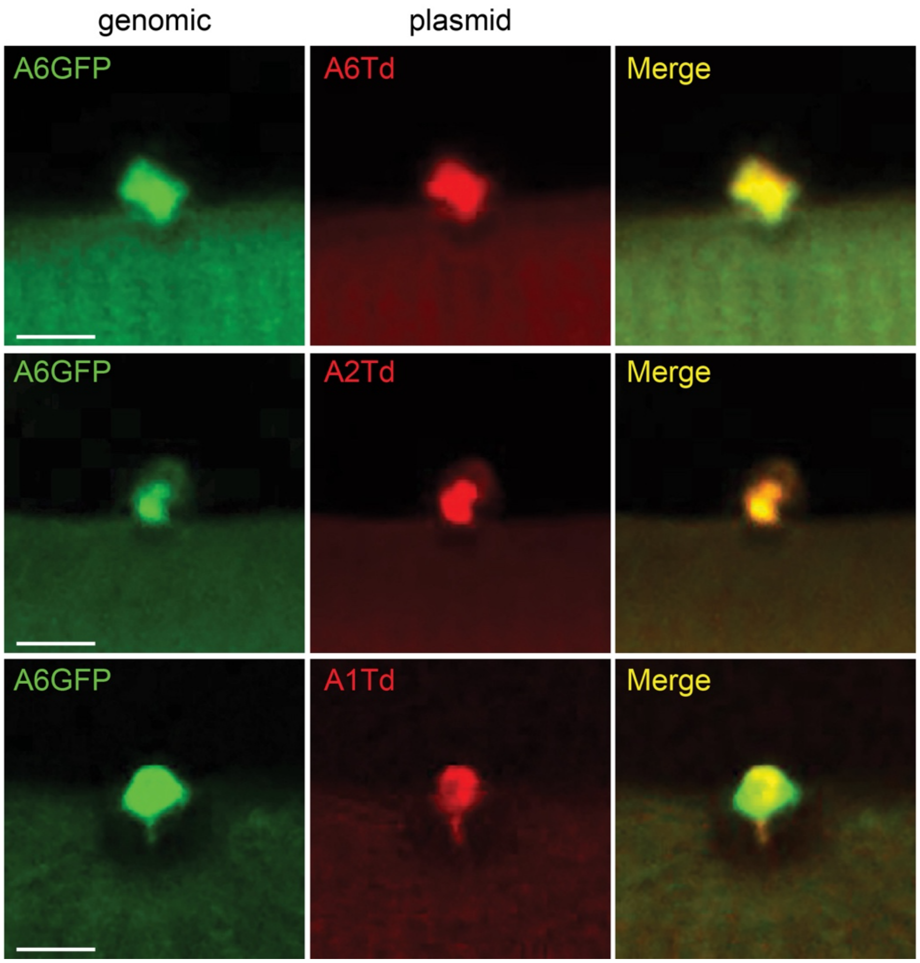

Supplemental Figure 2. Genomically-encoded annexin A6GFP colocalizes with repair complex members at the site of injury. Myofibers were isolated from heterozygous *Anxa6gfp* mice and electroporated with td-Tomato (red) tagged annexin A1, A2, or A6. Genomically-encoded annexin A6GFP (green) colocalizes with annexin A1, A2 and A6 at the site of membrane damage (merge, yellow). Z-stack images from an injured myofiber. Scale 5µm. $n \geq 7$ myofibers per condition.

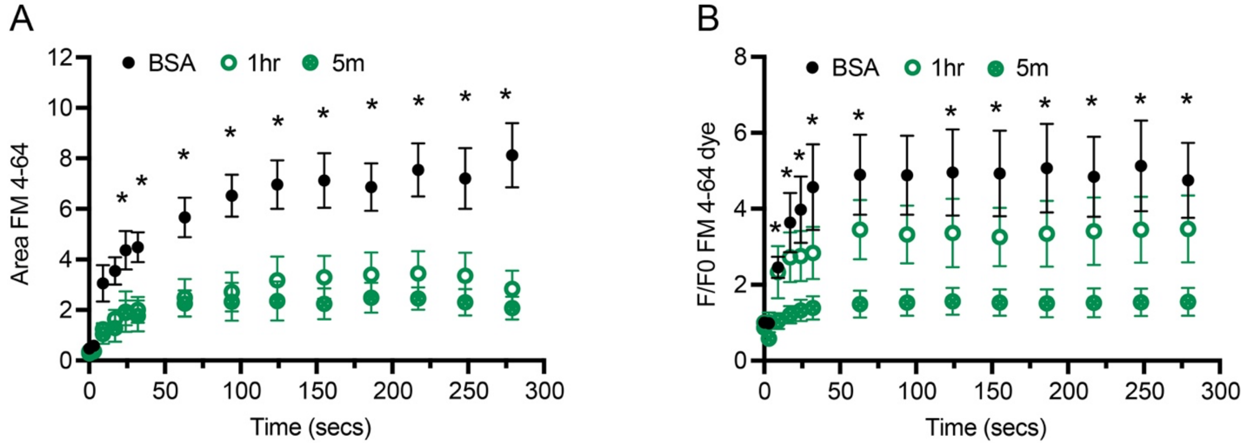

Supplemental Figure 3. Recombinant annexin A6 treatment reduces FM dye uptake. Quantification of FM 4-64 dye overtime showed that FM 4-64 was significantly reduced with 1hr and 5mins of recombinant annexin A6 treatment throughout the imaging series measured as (**A**) FM area or (**B**) fluorescent intensity. n = 6-8 myofibers from n ≥ 3 mice. * $P < 0.05$ by ANVOA.

Supplemental Movie Legends

Supplemental Movie 1. Z-stack imaging of genomically encoded annexin A6GFP (green) repair cap localized at the site of myofiber injury.

Supplemental Movie 2. Timelapse imaging of genomically encoded annexin A6GFP (green) localizing to the site of cardiomyocyte membrane injury over 50 seconds.

Supplemental Movie 3. Timelapse imaging of genomically encoded annexin A6GFP (green) localizing to the site of neuron membrane injury over 60 seconds.

Supplemental Movie 4. Timelapse imaging of recombinant annexin A6 tdTomato (red) colocalizing with genomically encoded annexin A6GFP (green) repair cap at the site of muscle membrane injury.

Supplemental Movie 5. Recombinant annexin A6 tdTomato (red) forms blebs at the site of myofiber injury.

Supplemental Movie 6. Timelapse imaging of recombinant annexin A6 tdTomato (red) localizing to the site of neuron transection. WGA outlines the neuron in blue.

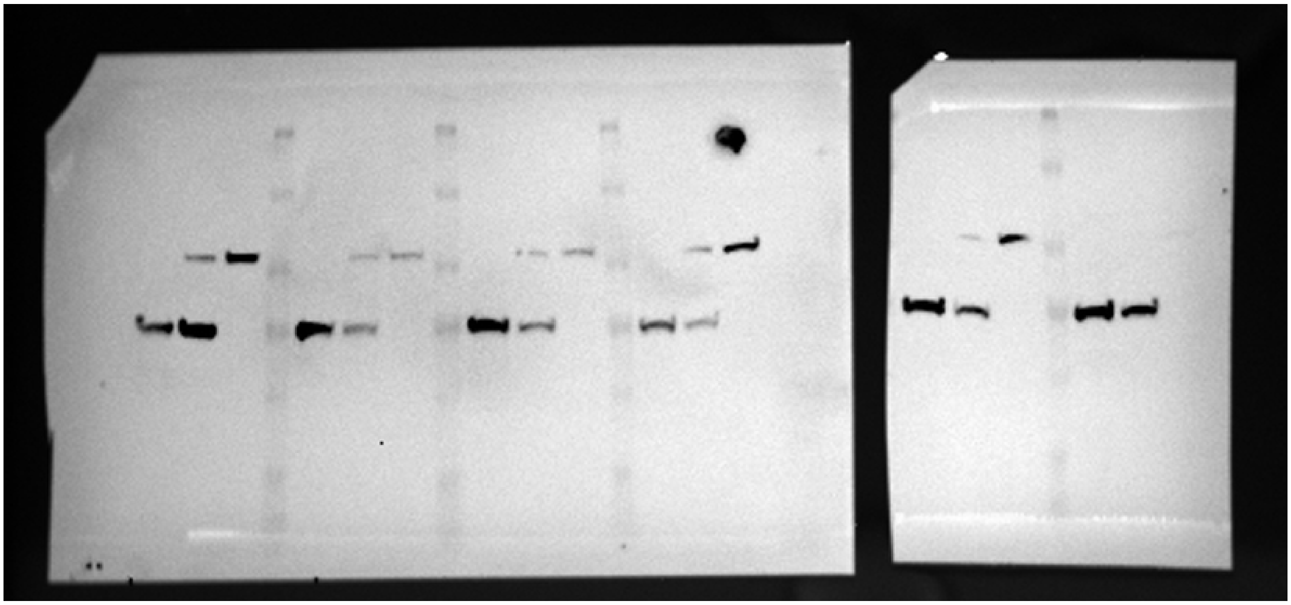

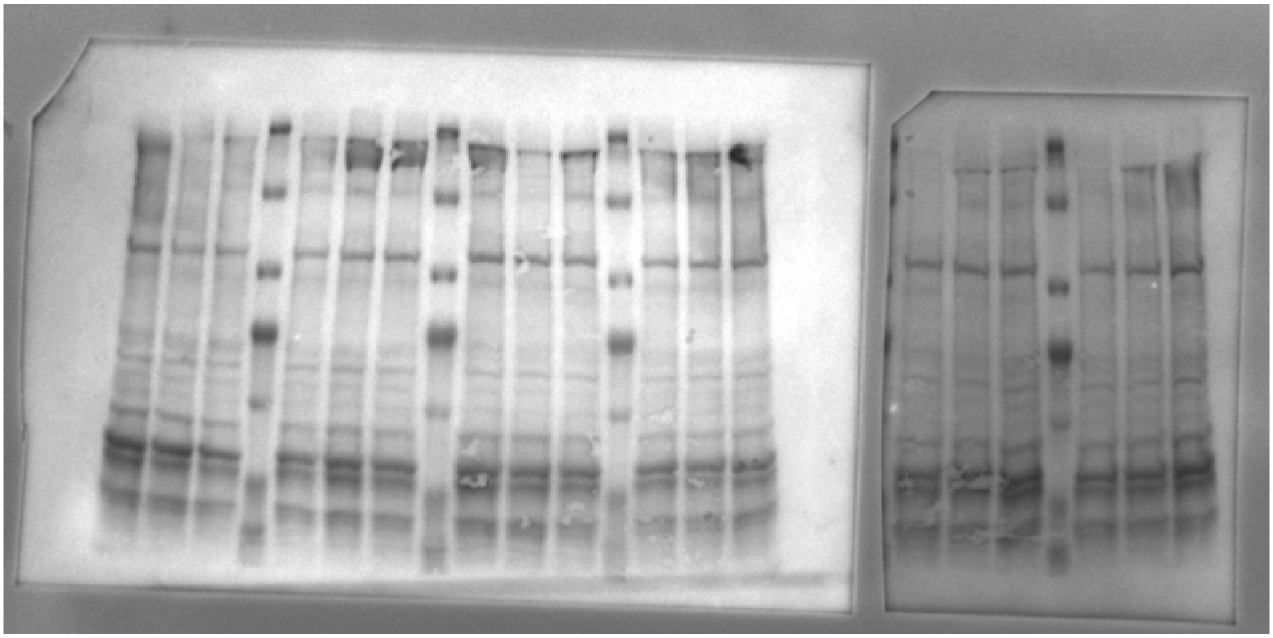

Full gels for Figure 2

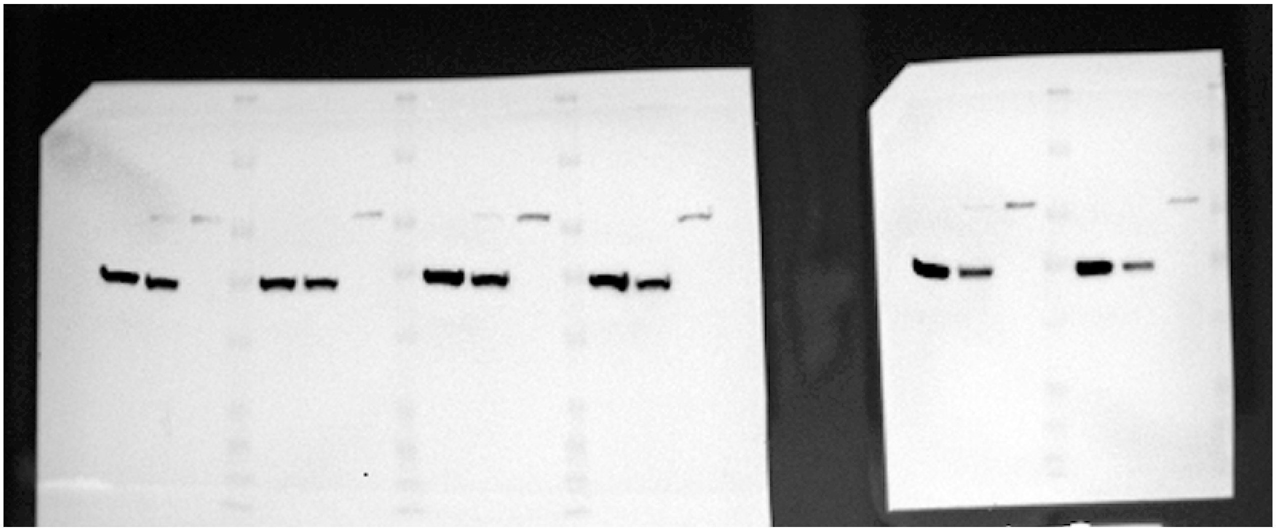

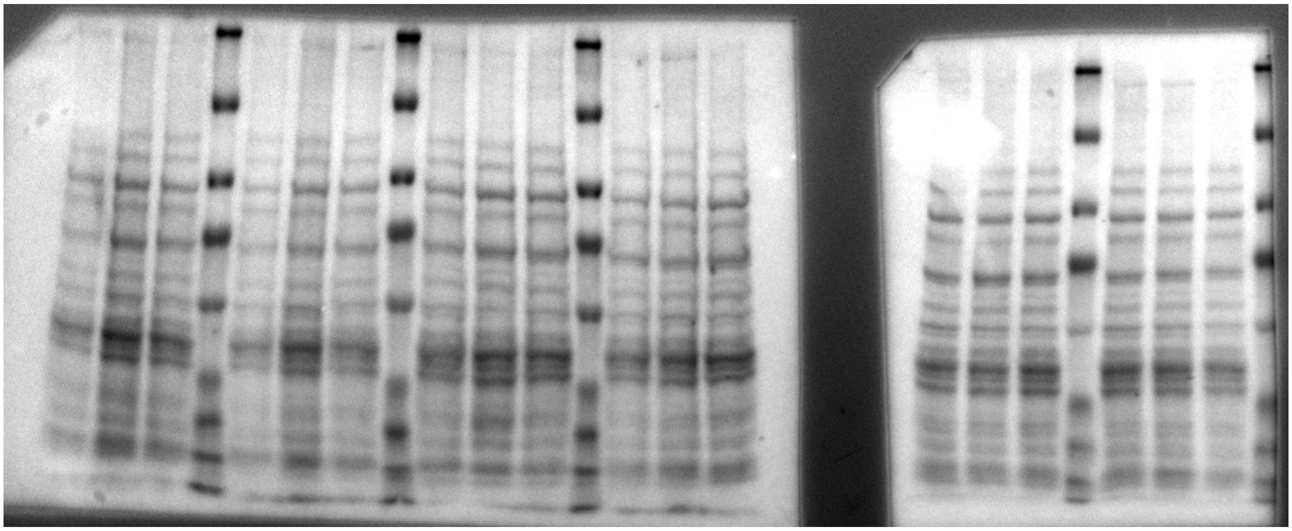

Full gels for Figure 3

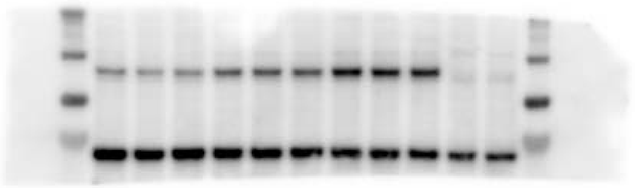

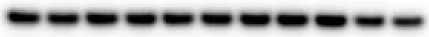

Full gels for Figure 4
